# A decadal analysis of bioeroding sponge cover on the inshore Great Barrier Reef

**DOI:** 10.1038/s41598-017-02196-z

**Published:** 2017-06-02

**Authors:** Blake D. Ramsby, Mia O. Hoogenboom, Steve Whalan, Nicole S. Webster, Angus Thompson

**Affiliations:** 1 College of Science and Engineering, James Cook University, Townsville, QLD Australia; 2 AIMS@JCU, Australian Institute of Marine Science and James Cook University, Townsville, QLD Australia; 3 Australian Institute of Marine Science, Townsville, QLD Australia; 4 ARC Centre of Excellence for Coral Reef Studies, James Cook University, Townsville, QLD Australia; 5 Marine Ecology Research Centre, School of Environment, Science and Engineering, Southern Cross University, Lismore, NSW Australia; 6 Australian Centre for Ecogenomics, University of Queensland, Queensland, QLD Australia

## Abstract

Decreasing coral cover on the Great Barrier Reef (GBR) may provide opportunities for rapid growth and expansion of other taxa. The bioeroding sponges *Cliona* spp. are strong competitors for space and may take advantage of coral bleaching, damage, and mortality. Benthic surveys of the inshore GBR (2005–2014) revealed that the percent cover of the most abundant bioeroding sponge species, *Cliona orientalis*, has not increased. However, considerable variation in *C*. *orientalis* cover, and change in cover over time, was evident between survey locations. We assessed whether biotic or environmental characteristics were associated with variation in *C*. *orientalis* distribution and abundance. The proportion of fine particles in the sediments was negatively associated with the presence-absence and the percent cover of *C*. *orientalis*, indicating that the sponge requires exposed habitat. The cover of corals and other sponges explained little variation in *C*. *orientalis* cover or distribution. The fastest increases in *C*. *orientalis* cover coincided with the lowest macroalgal cover and chlorophyll *a* concentration, highlighting the importance of macroalgal competition and local environmental conditions for this bioeroding sponge. Given the observed distribution and habitat preferences of *C*. *orientalis*, bioeroding sponges likely represent site-specific – rather than regional – threats to corals and reef accretion.

## Introduction

Loss of coral cover has led to dire predictions for the future of coral reef ecosystems^1–3^, including the Great Barrier Reef (GBR)^4^. A number of processes compromise coral health and the broader health of coral reefs, including increased sea surface temperatures, ocean acidification, pollution, cyclones, and crown of thorns starfish outbreaks^4, 5^. All of these stressors are predicted to intensify over coming decades, potentially shifting the coral reef benthic community from coral-dominated systems to those dominated by less-sensitive species^6, 7^. Some community changes have already been documented on coral reefs, including changes along acidification gradients at CO_2_ seeps^8^ and the octocoral and sponge dominance of shallow habitat of the Florida Keys, USA^9, 10^.

Changes to reef communities may reduce reef accretion, which represents the balance of calcification and consolidation with erosional processes^11^. Increased abundance of eroding organisms (bioeroders) or decreased abundance of calcifying organisms already suggest that some reefs are eroding rather than growing^12, 13^. Bioeroding sponges break down coral skeleton and other calcium carbonate structures, oyster shells, and cave walls. The sponges grow several mm to several cm into the coral skeleton and some species can quickly overgrow adjacent live coral tissue^14^. While sponges erode calcium carbonate at fast rates^15–17^, bioeroding sponges are patchily distributed, which currently limits their impact on regional carbonate budgets^13, 18^.

In some locations, bioeroding sponges (mostly *Cliona* spp.) have recently increased in abundance^19–22^. While these reports are largely restricted to single reefs, the rates of increase are notable: *Cliona caribbaea* cover doubled between 1979 and 1998 at one location in Belize^19^ and *Cliona* spp. abundance doubled between 1996 and 2001 in the Florida Keys, USA^21^. In addition, the abundance of *Cliona orientalis* more than doubled between 1998 and 2004 at one location in Queensland, Australia^20^. These changes gave rise to the hypothesis that the abundance of bioeroding sponges may be increasing over time, but the geographic extent and rate of these increases are largely unknown.

Several physiological and ecological hypotheses have been proposed to explain the observed increases in abundance of bioeroding sponges. *Cliona* is thought to be a robust sponge genus that is tolerant of disturbances and changing environmental conditions^23–26^ as well as benefitting from the poor water quality that can adversely affect corals^21, 27^. Based on the success of *Cliona* spp. in similar habitats, the inshore GBR was expected to be optimal habitat for bioeroding sponges where increases in cover may be occurring throughout the region^20^. However, poor water quality is also associated with low light conditions that may negatively impact growth of photo-symbiotic bioeroding sponges such as *C*. *orientalis* and *C*. *varians* ^28, 29^.

Increases in the abundance of bioeroding sponges will have implications for coral reefs in addition to the erosion of substratum^19^. Bioeroding sponges weaken reef substrata, produce carbonate sediments^11, 30, 31^, and are strong competitors against live corals^19, 32–37^, particularly following coral bleaching events^38^. However, the growth of *Cliona* spp. can be limited by macroalgae^32, 39^, suggesting that the composition of the reef community may influence the success of *Cliona*.

Given that sponge erosion is expected to accelerate as oceans become more acidic^18, 25, 40, 41^, there is a clear need to monitor bioeroding sponge populations^42, 43^. The most conspicuous bioeroding sponge on the GBR is *Cliona orientalis* but percent cover has only been reported for a single GBR site^20, 43, 44^. Here, we quantified the abundance and trajectory of *C*. *orientalis* cover on the inshore GBR over a 10-year period (2005–2014) to resolve whether environmental conditions are drivers of change in sponge abundance. Our sampling covers a wide geographic area to assess whether previous reports of increasing *Cliona* abundance represent a GBR-wide trend or site-specific responses^20^.

## Results and Discussion

**Figure 1:**
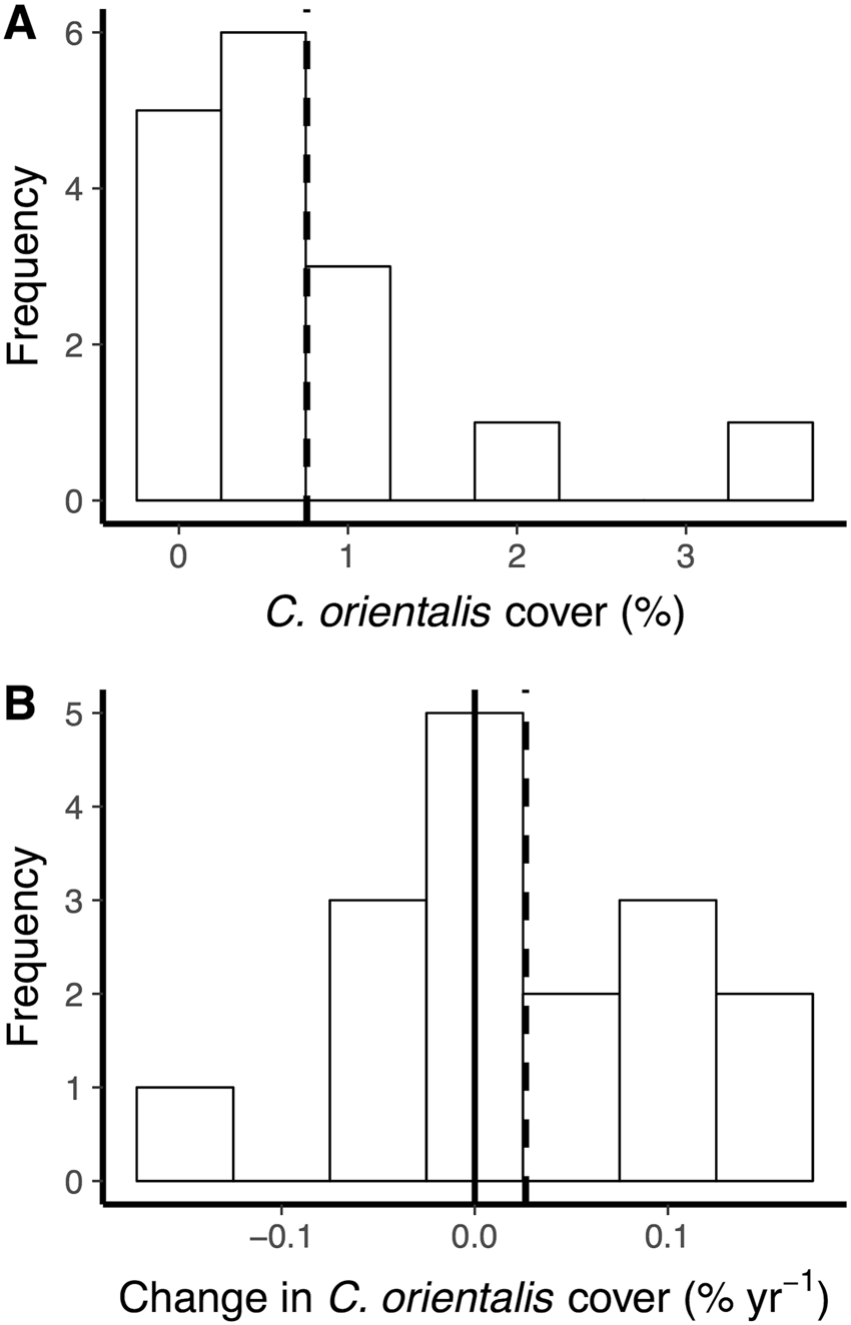
(**A**) Average percent cover of *Cliona orientalis* at the 16 sites used to measure changes over time. (**B**) Changes in *Cliona orientalis* cover per year. Changes in percent cover were estimated using linear regression and represent the average of 1–4 trends at each location. Dashed vertical lines indicate means and the solid vertical line indicates a value of zero.

**Figure 2:**
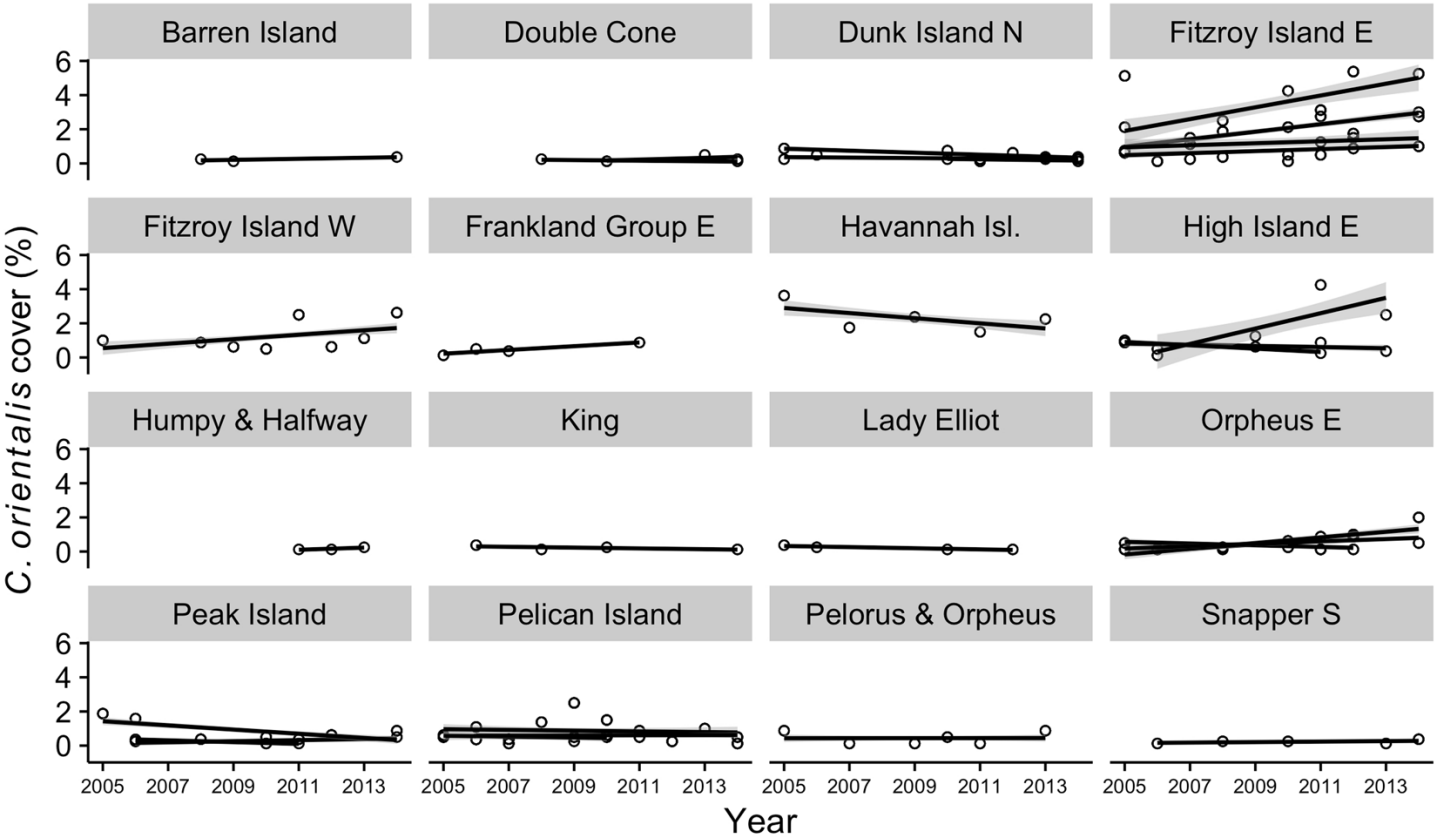
Trends in *Cliona orientalis* cover from 16 locations between 2005 and 2014. *C*. *orientalis* was found at 1–4 of the sites at each location. Linear regressions were fit to each site. Lines indicate the linear fit for each site and gray shading represents the standard error of the fit.

*C*. *orientalis* was present in at least three survey years at 16 of the 35 inshore GBR locations. Where present, *C*. *orientalis* occupied as much surface substratum (0.73% ± 0.97 SD) as all other sponges combined (0.56% ± 1.11 SD). Havannah Island had the highest average cover at 3.6% (Fig. 1A), although *C*. *orientalis* cover reached as high as 5% at Fitzroy Island and High Island in certain years (Fig. 2). *C*. *orientalis* percent cover was lower than previously reported from Orpheus Island (>6%)^20^, possibly due to a greater area surveyed or the untargeted design in the current study. When absences are included (i.e., zero cover), the average percent cover of *C*. *orientalis* on the inshore GBR was 0.14% (±0.51 SD), which is comparable to the average cover of *C*. *delitrix* in the Florida Keys, USA (~0.1%)^45^ and southeast Florida (~0.08%)^46^, but lower than *C*. *delitrix* cover in Colombia (~2%)^47^. Additional studies have assessed the abundance of bioeroding sponges by counting individual sponges^27, 38, 48, 49^, although it is challenging to reliably compare these measures of abundance with percent cover.

**Figure 3:**
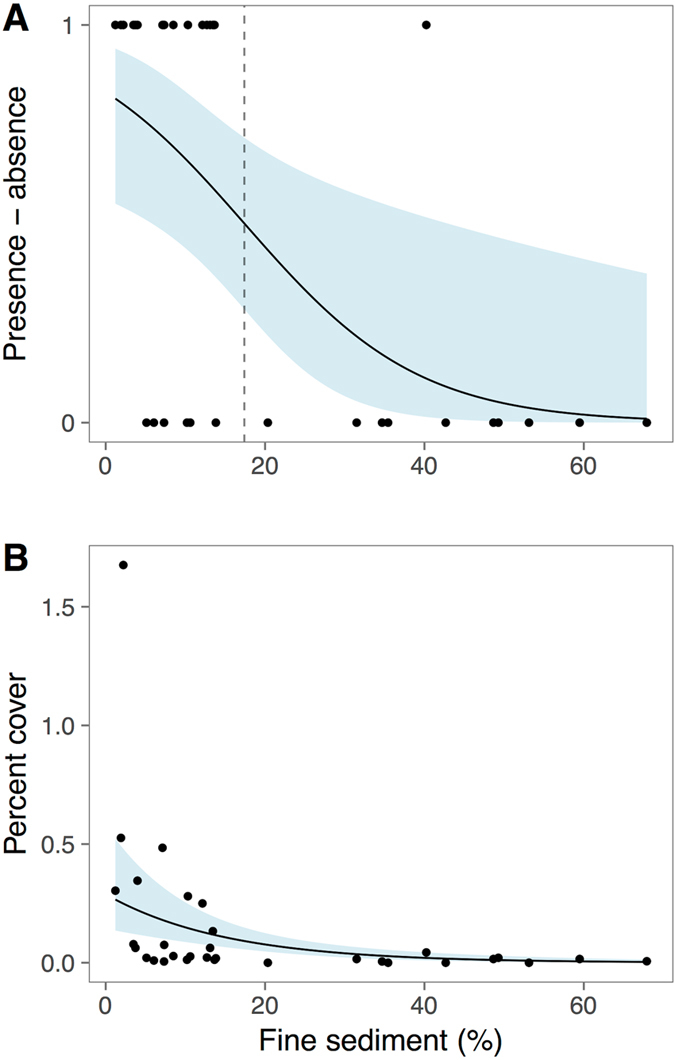
(**A**) *Cliona orientalis* was more likely to occur at sites with the lowest proportion of fine sediments (z = −2.5, P = 0.01; Wald test). The line represents the probability of occurrence using a binomial generalized linear model and shading represents the 95% confidence interval. The dashed line indicates the proportion of fine sediments (17%) with a 50% predicted probability of *C. orientalis* occurrence. Points represent presence-absence and the average fine sediment proportion for each location. (**B**) *Cliona orientalis* cover significantly decreased as a function of the proportion of fine particles in the sediment (z = −4.9, p < 0.01; Wald test). The line represents the predicted cover from a negative binomial generalized linear model and shading represents the 95% confidence interval. Points represent average cover and fine sediment proportion for each location.

*C*. *orientalis* occurred less frequently at locations with high accumulation of fine sediments. The model predicted a 50% probability of *C*. *orientalis* occurrence at 17% fine sediments, suggesting that even moderate accumulation of silt and clay sized particles prevents the establishment of *C*. *orientalis* (Fig. 3A). Furthermore, sites with large accumulations of fine sediments had low percent cover of *C. orientalis* (Fig. 3B). The amount of fine sediments distinguishes exposed and sheltered locations, as waves and currents resuspend fine particles and prevent accumulation^50^. Both suspended and deposited sediments can influence the composition of sponge communities^51^ and have negative physiological effects on sponges^52^, including reduced reproductive output^53^ and increased respiration^54^. The deposition of fine sediment may hinder filter-feeding or reduce the light available for photosynthesis^55^. The negative correlations observed between fine sediments and the distribution and abundance of *C*. *orientalis* suggest that sediments have negative physiological effects on *C*. *orientalis*, although these effects have not been demonstrated experimentally.

**Figure 4:**
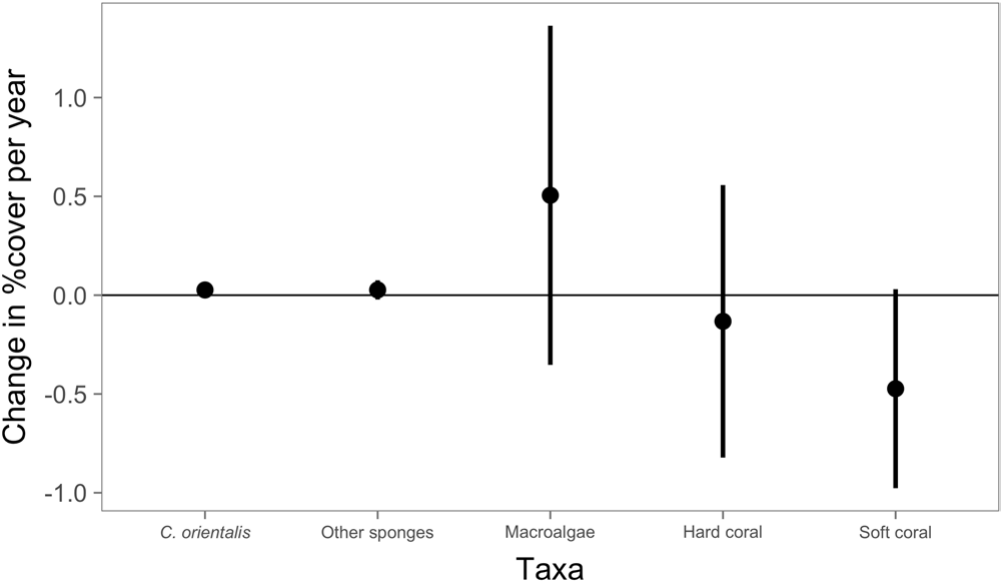
Changes in *Cliona orientalis* and sponge cover were near-zero, despite changes in other benthic groups. Points represent the average change in cover for the 16 locations where *C*. *orientalis* was present and error bars represent the 95% confidence interval.

As coral cover declines on the GBR^4^, changes in the cover of bioeroding taxa may dictate future reef growth^2, 13, 18^. In this study, the average change in *C*. *orientalis* percent cover was 0.03% yr^−1^ (±0.08 SD). Cover increased at 10 out of 16 locations (Fig. 1B), although only one trend was statistically significant (0.2% yr^−1^ at Fitzroy Island (East); t = 2.8, *p* < 0.05). *C*. *orientalis* cover exhibited non-linear patterns at some sites, possibly due to disturbances such as cyclones or outbreaks of crown of thorns starfish^56^, which altered community composition and potentially increased the detectability of *C*. *orientalis*. The rate of change in *C*. *orientalis* cover was similar to the rate of change in sponge cover at the same locations (0.03% yr^−1^ ± 0.10 SD), but slower than the changes in other benthic groups (Fig. 4). These time series indicate that cover of *C*. *orientalis* and other sponges has remained largely stable over the past decade on the inshore GBR despite changes to the reef community, such as a decline in octocoral cover (Fig. 4).

Few studies have reported the rate of change in percent cover of bioeroding sponges. Therefore, we estimated rates of change in cover of other *Cliona* spp. to provide context for the rates of change in *C*. *orientalis* cover measured in this study. The fastest estimated rate of change was for *C*. *orientalis* cover from 1998 to 2004 at Orpheus Island on the GBR (~0.9% yr^−1^)^20^. Slower rates of increase were reported from the Caribbean, where *C*. *caribbaea* cover increased ~0.14% yr^−1^ from 1979 to 1998 in Belize^19^, bioeroding sponge cover increased ~0.05% yr^−1^ from 2005 to 2009 in southwest Florida^57^ and *C*. *delitrix* cover changed <0.01% yr^−1^ from 2003 to 2009 in southeast Florida^46^. In contrast, *C*. *delitrix* cover decreased (−0.03% yr^−1^) in the Florida Keys^45^. The rate of change reported here (0.03% yr^−1^ for *C*. *orientalis*) is relatively low in the context of these estimates, but also encompassed a comparatively large number of survey locations. It is worth noting that many of the observations of increased cover of bioeroding sponges were initiated prior to 2001^19–21^ and that subsequent studies have not observed increased cover^38, 45, 46, 57^.

**Figure 5:**
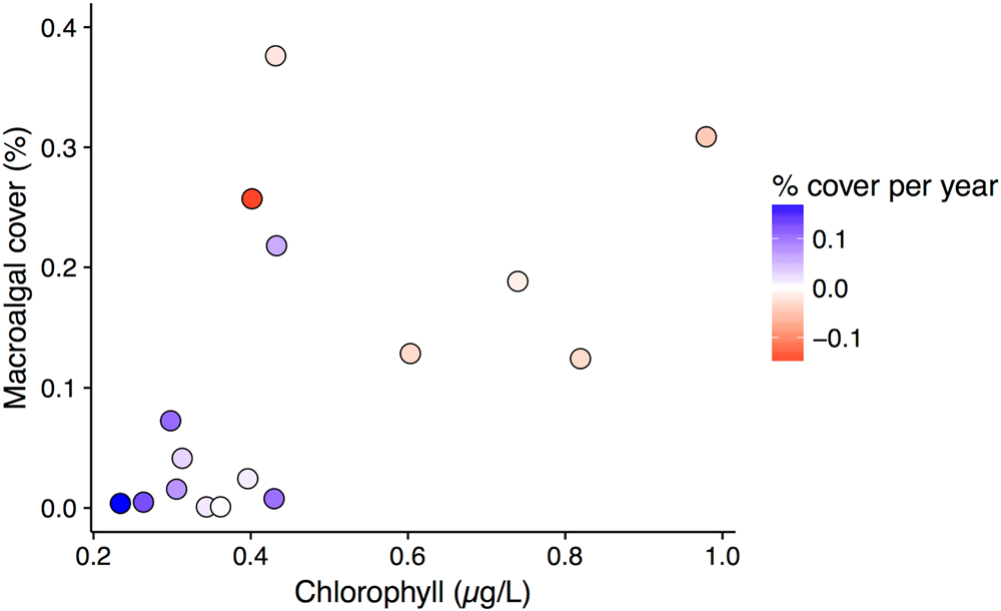
Change in *Cliona orientalis* cover was highest at locations with low chlorophyll concentration and low macroalgal cover. Points represent average macroalgal cover and chlorophyll *a* concentrations for each location and the color indicates the direction of change in *C*. *orientalis* cover.

**Figure 6:**
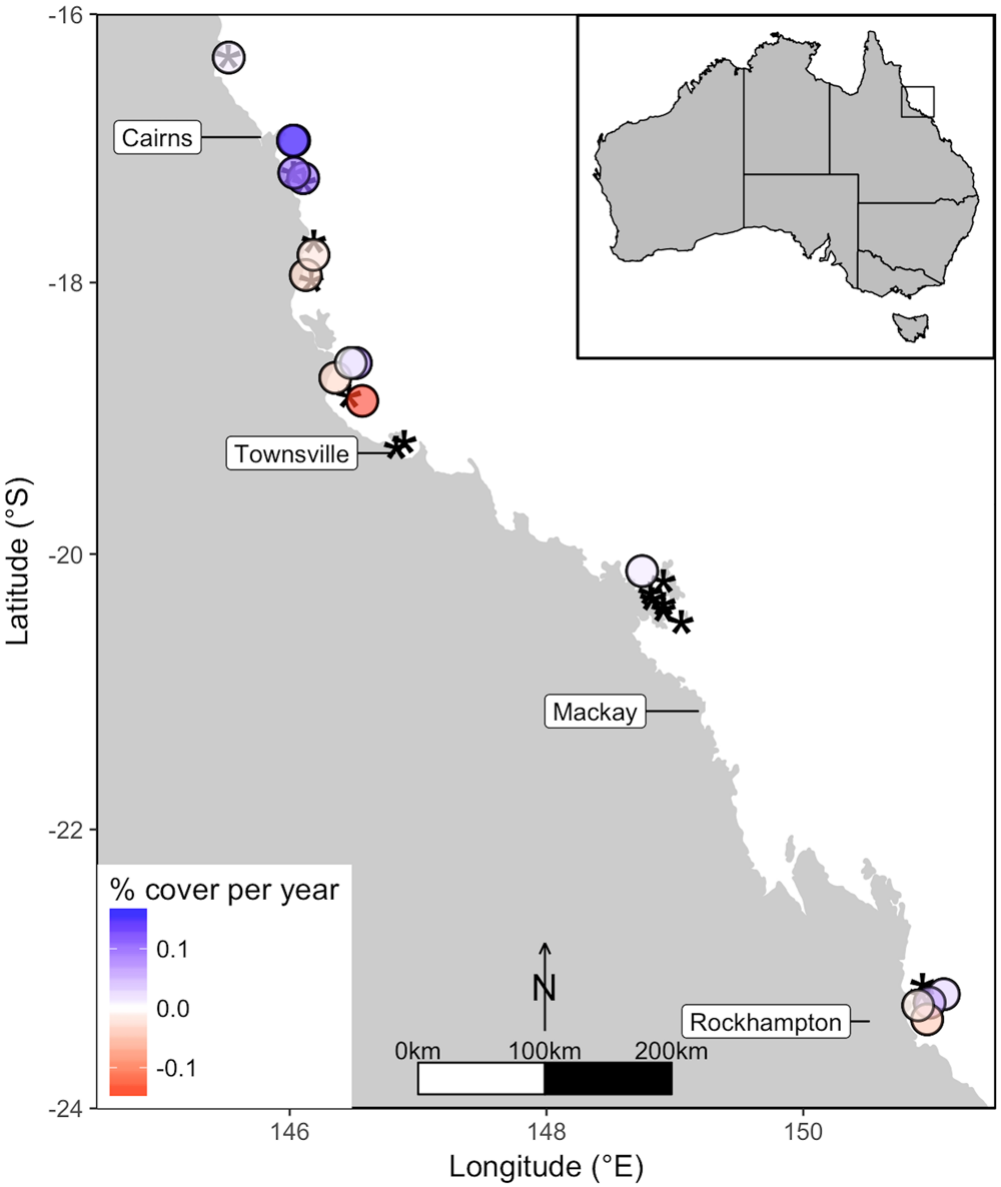
The spatial distribution of changes in *C*. *orientalis* cover on the inshore GBR. Circles represent locations where change over time was measured and an * represent locations where *C*. *orientalis* was absent (not detected in at least 3 survey years). Blue circles indicate increases in cover; white circles indicate zero change in cover; and red circles indicate decreases in cover. The map was created using R statistical software (version 3.3.1; https://www.r-project.org) and the packages “ggplot2”^69^, “mapdata”^70^, and “oz”^71^.

Changes in *C*. *orientalis* cover are best explained by the abundance of macroalgae (Fig. 5, Table 1). Increases in *C*. *orientalis* cover occurred at locations with low macroalgal cover (t = −3.0, P = 0.01). However, these locations also had low average chlorophyll *a* concentration in the water (Fig. 5), which also significantly affected the change in *C*. *orientalis* cover (t = −2.4, P = 0.03). Therefore, the fastest increases in *C*. *orientalis* cover occurred at locations with a combination of low macroalgal cover and low chlorophyll concentrations, which were clustered near Cairns (Fig. 6). When analysed together, neither macroalgal cover nor chlorophyll concentration was significantly associated with change in *C*. *orientalis* cover (P > 0.05), likely due to the positive correlation between macroalgal cover and chlorophyll concentration (r = 0.56). While macroalgal cover explained 39% of the variation in change in *C*. *orientalis* cover (Table 1), macroalgal cover (or chlorophyll *a*) did not predict the distribution or abundance of *C*. *orientalis* (Table 1, Supplementary Figure 1).

**Table 1:** Environmental variables are stronger predictors of *Cliona orientalis* distribution and abundance than biotic variables.

Response	Predictors			R^2^	AIC	∆AIC
	Category	n	Description			
Presence-absence	Environmental	3	Chlorophyll *a*, fine sediment*, total carbon in sediment	0.29	39.5	3.4
		1	Chlorophyll *a*	0.01	47.8	11.7
		1	Total carbon in sediment	0.12	43.1	10.0
		**1**	Fine sediment*	**0.28**	**36.1**	**0**
	Biotic	4	Coral, macroalgae, sponge, and abiotic percent cover	0.11	44.2	7.9
	Geography	1	Latitude	0	52.1	16.0
Percent cover	Environmental	3	Chlorophyll *a*, fine sediment*, total carbon in sediment	0.40	260.0	36.0
		**1**	Fine sediment*	**0.38**	**256.8**	**32.8**
		1	Total carbon in sediment*	0.19	266.6	42.6
		1	Chlorophyll *a*	0	275.7	51.7
	Biotic	4	Coral, macroalgae, sponge, and abiotic percent cover	0.10	224.0	0
	Geography	1	Latitude	0.05	272.9	48.9
Change in percent cover	Environmental	3	Chlorophyll *a*, fine sediment, total carbon in sediment	0.33	−32.6	5.4
		1	Chlorophyll a*	0.30	−35.9	2.1
		1	Total carbon in sediment	0.12	−32.3	5.7
		1	Fine sediment	0.03	−30.7	7.3
	Biotic	4	Coral, macroalgae*, sponge, and abiotic percent cover	0.42	−33.0	5.0
		1	Coral	0.01	−30.4	7.6
		**1**	Macroalgae*	**0.39**	**−38.0**	**0**
		1	Sponge	0.05	−31.0	7.0
		1	Abiotic	0.11	−32.1	5.9
	Geography	1	Latitude	0.10	−31.8	6.2

The table contains a comparison of models with three categories of predictors, representing the hypotheses that the *C*. *orientalis* response was influenced by the percent cover of other taxa, environmental conditions, or latitude. The table includes the *C*. *orientalis* response variable; the category, number and description of predictors; the proportion of deviance explained by the predictors (R^2^); and the Aikaike Information criterion score (AIC). An * indicates predictors which were statistically significant and statistics are reported in figure legends. The most parsimonious model, in terms of R^2^ and AIC, is indicated in bold.

These results suggest that macroalgae outcompete bioeroding sponges for space: all but one of the locations with increased *C*. *orientalis* cover had less than 10% macroalgal cover and all had less than 0.45 µg/L chlorophyll *a* (Fig. 5), a water quality threshold that separates reefs with low and high macroalgal abundance^58^. Previous work observed that macroalgal cover was negatively correlated with *C*. *orientalis* cover^39^ and macroalgae have also been reported to outcompete *C*. *tenuis* for substratum in the Caribbean^32^. In addition, several studies have observed that large colonies of bioeroding sponges occur where macroalgal cover is low^32, 59^. By extension, controls on macroalgal growth, such as fish and urchin herbivory^39^ as well as dissolved nutrient levels^58^, may indirectly affect the growth of bioeroding sponges.

The gradual increases in *C*. *orientalis* cover observed at multiple locations suggest that broader ecological changes may be responsible for increases in *C*. *orientalis* cover. Water quality is declining across the inshore GBR, driven by inputs of terrestrial nutrients that are delivered during seasonal flood events^60, 61^. Dissolved nutrient levels increase during floods^61^, which can lead to phytoplankton blooms and higher concentrations of organic material in the water^62^, which is a primary food for some *Cliona* species^63^. Nutrient levels likely increased over the survey period, as river flows were high, particularly during the middle of the study^56, 64^. At locations with high nutrient levels, additional nutrients would likely have benefited the already high macroalgal cover^58^. However, at locations with low nutrient levels and little cover of macroalgae, additional nutrients may have contributed to increases in *C*. *orientalis* cover (Fig. 5). Thus, increases in *C*. *orientalis* cover may reflect additional nutrient loads entering the GBR lagoon, but are restricted to locations where nutrient concentrations are insufficient to support high macroalgal cover.

While the response of *C*. *orientalis* to high nutrient levels has not been investigated experimentally, several other *Cliona* species exhibit positive associations with elevated nutrients, including *C*. *delitrix* and *C*. *vastifica* ^21, 27, 47, 65^. However, not all *Cliona* species respond the same way, as several exhibited either positive or negative responses to a chlorophyll *a* gradient in Mexico^48^. On the GBR, observation of higher abundance of bioeroding sponges on inshore versus offshore reefs suggests that bioeroding sponges benefit from high nutrient conditions^66^. The correlations reported here suggest that *C*. *orientalis* is affected by local environmental conditions, specifically fine sediments, dissolved nutrients (chlorophyll *a*), and macroalgal cover, but experimental evidence of how these conditions affect *Cliona* species is lacking.

Factors other than fine sediments, macroalgal cover, and chlorophyll *a* explained little variation in the cover or distribution of *C*. *orientalis*. The cover by other taxa (scleractinian corals, soft corals, sponges, macroalgae) did not influence the distribution or abundance of *C*. *orientalis* (Table 1; Supplementary Figure 1), suggesting that competition with these groups does not exclude *C*. *orientalis* from its habitat. Total carbon in the sediment explained some variation in *C*. *orientalis* abundance, but the effect was not significant in a model that included both total carbon and fine sediments (Table 1). Latitude explained little variation in *C*. *orientalis* abundance or distribution (Table 1) or in the environmental predictors (Supplementary Figure 2). Importantly however, processes affecting *C*. *orientalis* at small spatial scales were not accounted for. For example, whilst the presence-absence of *C*. *orientalis* varied between nearby locations (i.e., kilometres; Fig. 6), presence-absence also varied within locations (i.e., 250 m). Much of the unexplained variation in the distribution and cover of *C*. *orientalis* may be due to small-scale factors, such as the availability of hard substratum^44^.

## Conclusion

Here, we present a large-scale monitoring effort to assess temporal changes in the abundance of the bioeroding sponge *Cliona orientalis* on the inshore GBR. Whilst *Cliona* abundance increased at 11 of 16 locations, increases in macroalgal cover and decreases in scleractinian and octocoral cover all outpaced changes in *Cliona* abundance. Low deposition of fine sediments was strongly associated with both the presence and abundance of *C*. *orientalis*, suggesting that the sponge requires exposed habitat. Increased cover of *C*. *orientalis* was only observed where mean chlorophyll *a* concentration was less than 0.45 µg/L and macroalgal cover was low, suggesting that *C*. *orientalis* can only increase in habitats where macroalgae are nutrient-limited. Experimental work that identifies the limiting environmental conditions (light, suspended sediment, nutrients) for *C*. *orientalis* is clearly warranted. Given the clumped distribution and strong association with local environmental conditions (e.g., sediment, macroalgae), bioeroding sponges such as *C*. *orientalis* likely represent site-specific – rather than regional – threats to coral health and reef accretion on the GBR.

## Methods

### Benthic surveys

Benthic cover was surveyed at 35 locations on the inshore GBR between 2005 and 2014 as part of the Inshore Water Quality and Coral Reef Monitoring program at the Australian Institute of Marine Science^56^. Briefly, at each location, two sites and two depths (2 and 5 m) were surveyed using five, fixed, 20 m transects. Every 0.5 m along each transect, photographs were taken of the benthos, which were used to determine presence-absence, percent cover, and change in percent cover. Survey data were pooled across sites and depths to relate to environmental variables measured at each location.

Percent cover was measured from digital photographs of the benthos. Five markers were overlaid onto each photograph and percent cover was calculated as the proportion of points occupied by each taxon. Percent cover of *C*. *orientalis* (encrusting ß form), other sponges, scleractinian corals, octocorals, and macroalgae was calculated for each of the four within-location survey sites. The influence of other benthic taxa on *C*. *orientalis* cover was explored using biplots of cover at each within-location site.

Trends in cover were analysed for each within-location site where *C*. *orientalis* was detected in at least three survey years. Trends were estimated for each location separately as the locations were surveyed at different frequencies over the course of the study. Change in percent cover was estimated for each within-location site using linear regression. Thus, change in percent cover represents the average of the within-location sites (1–4) where *C*. *orientalis* was detected. Analysis of presence-absence of *C*. *orientalis* at each location followed the same criterion as change in percent cover, whereby *C*. *orientalis* was considered present if it occurred in at least three survey years at any of the sites.

### Environmental variables

Survey data were related to environmental variables collected at the location scale (not sites or transects). Water quality was assessed using satellite-derived data from the eReefs Marine Water Quality Dashboard (http://ereefs.org.au/ereefs), including chlorophyll *a* concentration, coloured dissolved organic matter, and non-algal particulates (1 km resolution). The data nearest each survey location were analysed for each survey year. Sediment was collected from the 5 m survey sites and the proportion of fine particles, carbon content, and nitrogen content in the sediment were measured as described in^56^, with average values compared to *C*. *orientalis* cover. Fine particles in the sediments were defined as all particles smaller than 63 µm and expressed as a proportion of the total sediment^67^.

### Data analysis

Exploratory plots were prepared to identify correlations amongst the environmental predictors and to compare the effects of different benthic taxa on *C*. *orientalis* cover. Note that only fine sediment, chlorophyll *a*, and total C in sediment were included in the model, as other environmental variables were strongly correlated with either the proportion of fine sediments or chlorophyll *a* (all r > 0.7). Uncorrelated environmental variables were used to predict the presence-absence of *C*. *orientalis* (generalized linear model (GLM) with binomial errors and logit link), the percent cover of *C*. *orientalis* (GLM with negative binomial errors and log link), and changes in *C*. *orientalis* cover per year (linear model). Latitude was used to account for the spatial relationships among locations. Model fit was evaluated by plotting residual and fitted values. For generalized linear models, model fit was also evaluated using the chi-square probability of the residual deviance and residual degrees of freedom and by comparing observed and simulated residuals from each model.

Three models were used to assess whether other taxa, the environment, or geography explained patterns in *C*. *orientalis* distribution. Models were compared using AIC and R^2^ values. For the GLM, R^2^ was calculated as the deviance ratio of models with and without predictors. The most parsimonious model was identified as the model that maximized explanatory power with the fewest predictors.

Analyses were conducted in R statistical software^68^. The map in Fig. 6 was produced using R statistical software and the packages, “ggplot2”^69^, “mapdata”^70^, and “oz”^71^ packages.

## Electronic supplementary material

Supplementary Information
