# Supplementary material for: A decadal analysis of bioeroding sponge cover on the inshore Great Barrier Reef
Source: Sci Rep. 2017 Jun 2;7:2706. doi: 10.1038/s41598-017-02196-z (PMC5457454; doi:10.1038/s41598-017-02196-z)

A decadal analysis of bioeroding sponge cover on the inshore Great Barrier Reef

Blake D. Ramsby[1, 2, 3], Mia O. Hoogenboom[1, 4], Steve Whalan[5], Nicole S. Webster[3, 6], and Angus Thompson[3]

1 College of Science and Engineering, James Cook University, Townsville, QLD, Australia.

2 AIMS@JCU, Australian Institute of Marine Science and James Cook University, Townsville, QLD, Australia.

3 Australian Institute of Marine Science, Townsville, QLD, Australia.

4 ARC Centre of Excellence for Coral Reef Studies, James Cook University, Townsville, QLD, Australia

5 Marine Ecology Research Centre, School of Environment, Science and Engineering, Southern Cross University, Lismore, NSW, Australia

6 Australian Centre for Ecogenomics, University of Queensland, QLD, Australia

Supplementary Figure 1

The relationship between *C. orientalis* cover and the cover of other taxa is similar for algae, scleractinian corals, soft corals, and other sponges. Points represent the average cover for all survey sites (within each location) where *C. orientalis* is present.

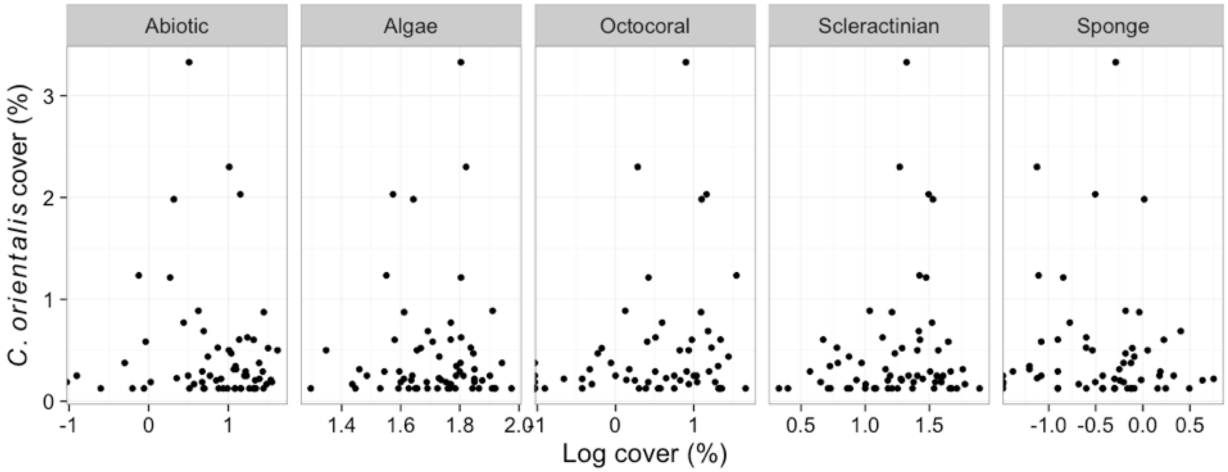

Supplementary Figure 2

Relative chlorophyll (open circles) and fine sediment (closed circles) as a function of latitude. Chlorophyll was similar across latitudes, but sediment values were highest at intermediate latitudes.

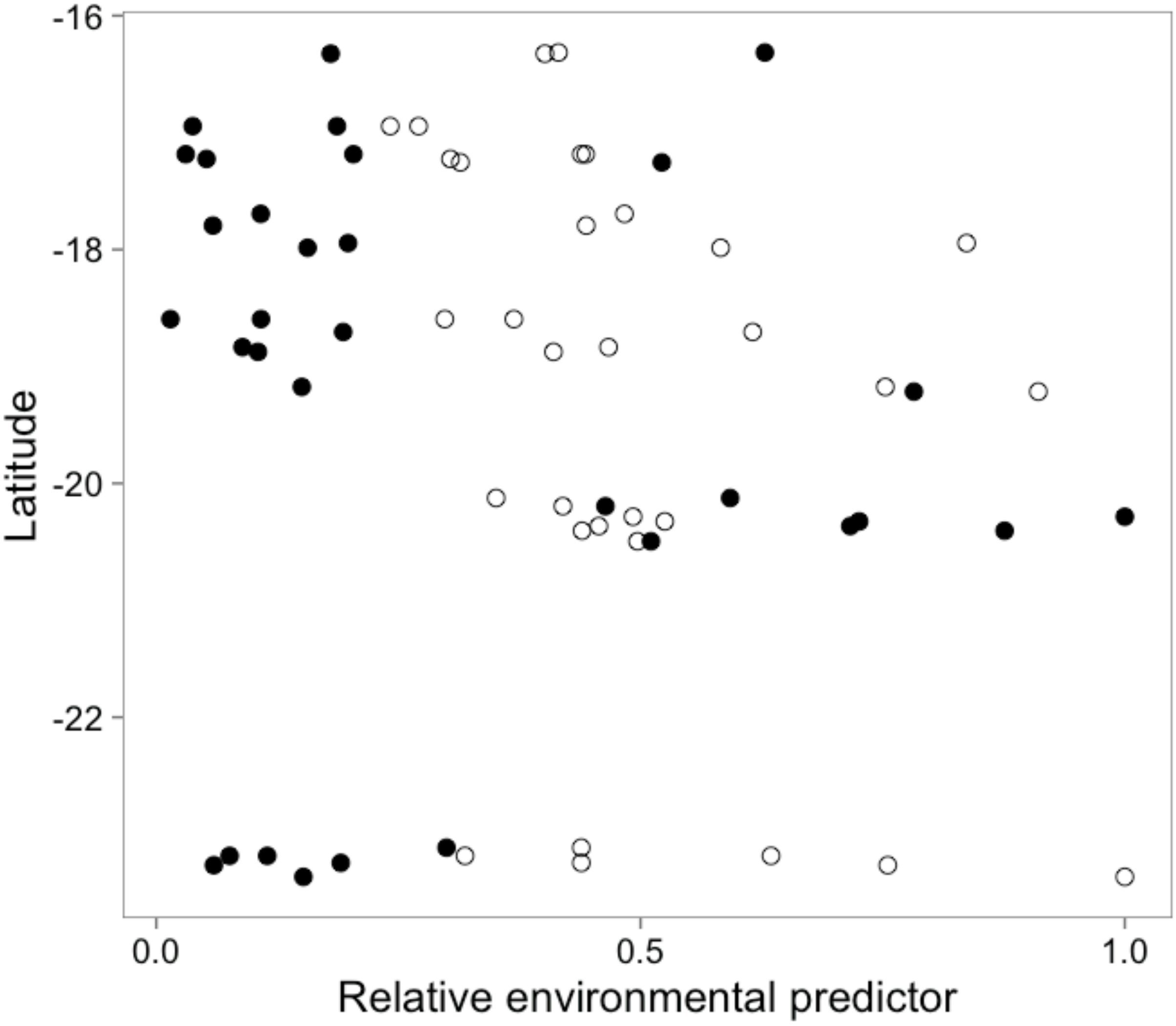

Supplement: Supplementary file 1 — Supplementary Information [file 41598_2017_2196_MOESM1_ESM.pdf]
